# Artificial intelligence models using F-wave responses predict amyotrophic lateral sclerosis

**DOI:** 10.1093/brain/awaf014

**Published:** 2025-01-16

**Authors:** Jennifer M Martinez-Thompson, Kevin A Mazurek, Carolina Parra-Cantu, Elie Naddaf, Venkatsampath Gogineni, Hugo Botha, David T Jones, Ruple S Laughlin, Leland Barnard, Nathan P Staff

**Affiliations:** Department of Neurology, Mayo Clinic, Rochester, MN 55905, USA; Department of Neurology, Mayo Clinic, Rochester, MN 55905, USA; Department of Neurology, Washington University at St. Louis, St. Louis, MO 63110, USA; Department of Neurology, Mayo Clinic, Rochester, MN 55905, USA; Department of Neurology, Mayo Clinic, Rochester, MN 55905, USA; Department of Neurology, Mayo Clinic, Rochester, MN 55905, USA; Department of Neurology, Mayo Clinic, Rochester, MN 55905, USA; Department of Neurology, Mayo Clinic, Rochester, MN 55905, USA; Department of Neurology, Mayo Clinic, Rochester, MN 55905, USA; Department of Neurology, Mayo Clinic, Rochester, MN 55905, USA

**Keywords:** amyotrophic lateral sclerosis, artificial intelligence, electrodiagnostic, machine learning, prediction, survival

## Abstract

Nerve conduction F-wave studies contain crucial information about subclinical motor dysfunction that can be used to diagnose patients with amyotrophic lateral sclerosis (ALS). However, F-wave responses are highly variable in morphology, making waveform interpretation challenging. Artificial intelligence techniques can extract time–frequency features to provide new insights into ALS diagnosis and prognosis.

A retrospective analysis was performed on F-wave responses from 46 802 patients. Discrete wavelet transforms were applied to time-series waveform responses after stimulating ulnar, median, fibular and tibial nerves. Wavelet coefficient statistics, onset age, sex and body mass index were features for training a Gradient Boosting Machine model on 40 095 patients (5329 diagnosed with motor neuron disease). Model performance was tested on responses from 689 ALS patients meeting Gold Coast criteria and 689 age- and sex-matched controls. An exploratory analysis examined model performance on cohorts of patients with inclusion body myositis, cervical radiculopathy, lumbar radiculopathy or peripheral neuropathy, which can mimic ALS symptoms. Factors affecting survival were estimated through Cox proportional hazards regression.

The model trained using wavelet features on the full waveform had 90% recall, 87% precision and 88% accuracy. Similar model performance was measured using features from only the M-wave or F-wave. Classification probabilities for ALS patients were statistically different from the diagnoses mimicking ALS symptoms (*P* < 0.001, ANOVA, Tukey’s *post hoc* test). Higher model classification probabilities of ALS, older age at onset, and family history of ALS alone or with frontotemporal dementia were factors decreasing survival. Longer diagnostic delay and upper limb onset site were factors increasing survival. Model scores two standard deviations below the mean had 4 months increased survival (two standard deviations below had 3 months decreased survival).

Artificial intelligence techniques extracted important information from F-wave responses to estimate a patient’s likelihood of ALS and their survival risks. Although the model can make predictions at a specific decision threshold as presented here, the true strength of such a model lies in its ability to provide probabilities about whether a patient is likely to have ALS in comparison to other mimicking diagnoses, such as inclusion body myositis, cervical or lumbar radiculopathy or peripheral neuropathy. These probabilities provide clinicians with additional information that they can use to make the final diagnosis with greater confidence and precision. Integrating such a model into the clinical workflow could help clinicians to diagnose ALS sooner and manage treatment based on estimated survival, which might improve outcomes and the quality of life of patients.

## Teaser

ALS is a complex disease that presents challenges for early diagnosis. Martinez-Thompson *et al.* show that AI approaches applied to clinical electromyography data can assist in diagnosing ALS and estimating patient survival. These models have the potential to help clinicians make earlier diagnoses and improve disease management.

## Introduction

Amyotrophic lateral sclerosis (ALS) is a progressive, fatal paralytic neurodegenerative disorder of motor neurons with an average survival of 2–5 years after symptom onset.^1-4^ Despite the lack of curative treatments, early diagnosis of ALS can improve clinical outcomes and quality of life for affected individuals and their caregivers.^5^ Early diagnosis also allows for enrolment into clinical trials at a disease stage when investigational products might be most effective.^6^ A challenge in ALS diagnosis is the variability in the neuroanatomical region of onset and rate of disease progression, which can lead to diagnostic delays and ambiguity in prognostication after diagnosis. Electrodiagnostic studies are often performed to determine sources of motor dysfunction. Artificial intelligence (AI) models could be trained on features extracted from these electrodiagnostic studies to improve the efficacy with which ALS is diagnosed and managed.

**Figure 1:**
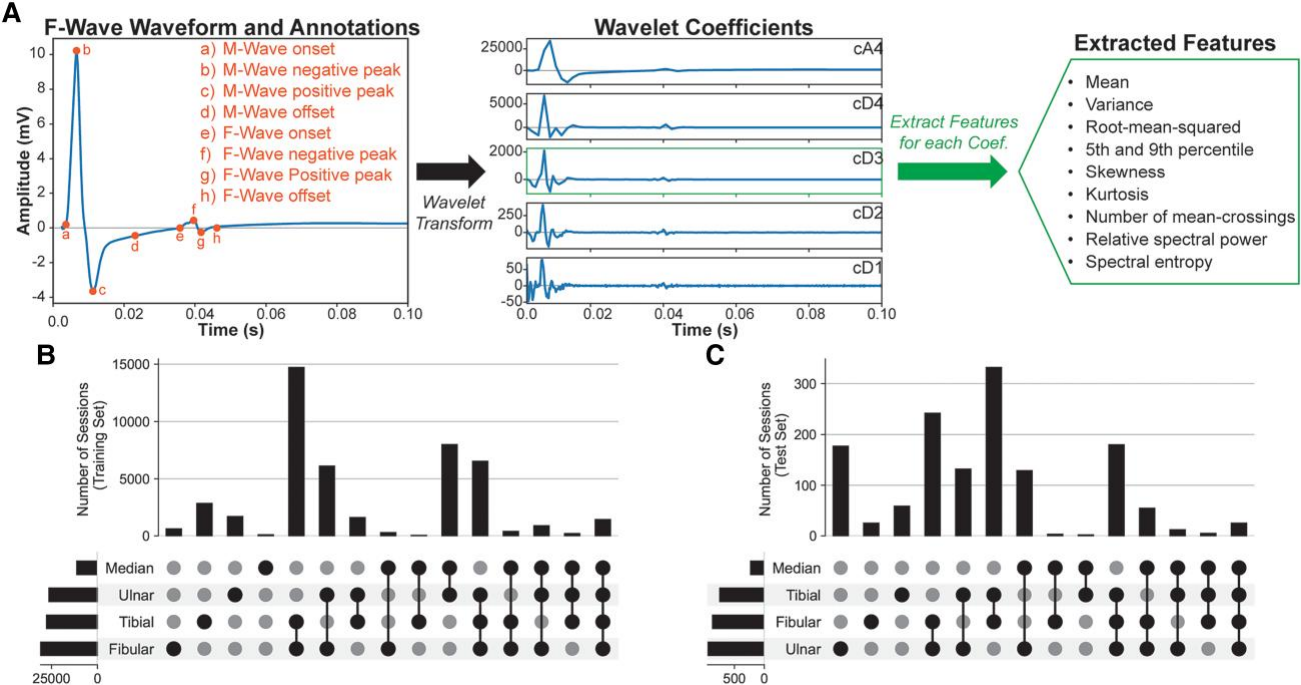
**A depiction of the F-wave waveform, wavelet transformation and breakdown of the number of nerves stimulated by session.** (**A**) Schematic diagram of the F-wave waveform, wavelet transform and feature extraction. Some F-wave waveforms have clinical annotations to detect peaks, onset and offset of the M-wave and F-wave. Five wavelet coefficients were calculated and represented as cA4 and cD4–cD1. Features were extracted for each wavelet coefficient (the *top*  *right* represents one set of coefficients). (**B** and **C**) UpSet plot indicating the number of sessions in which each combination of nerves was stimulated in the training set (**B**) and the test set (**C**). The horizontal bar graph to the *left* indicates the total number of sessions in which a nerve was stimulated. Vertical bar graphs on *top* indicate the number of sessions in which the different combinations of nerves were stimulated.

Electrodiagnostic studies are used routinely in the diagnosis of neuromuscular diseases, including ALS.^2,7^ One such procedure is the F-wave study, in which a nerve is stimulated to evoke compound muscle action potentials. The first potential (M-wave) is the result of orthodromic activation of the nerve to the recording muscle, followed by a second potential (F-wave) resulting from antidromic activation of anterior horn cells in the spinal cord (Fig. 1A).^8,9^ The F-wave response helps to determine conduction along the proximal course of a motor nerve, which can help to infer the health of anterior horn cells impaired in ALS or proximal motor nerve segments in peripheral nerve disorders that mimic ALS but require different management and prognostic considerations.^10^ Higher amplitudes and increased chronodispersion have been reported in the F-wave potential from ALS patients compared with controls.^2^ Numerous studies have attempted to create biomarkers based on specific features of the F-wave study response. The Neurophysiological Index (NI) calculates the ratio of the M-wave amplitude and the distal motor latency and scales this by the fraction of times an F-wave response is observed.^11,12^ The NI is correlated with muscle strength and longitudinal disease progression.^13-17^ However, the NI still requires detection of the F-wave response, which can be challenging, depending on the rate of disease progression.^14^ Similar to other EMG approaches,^18,19^ time–frequency analyses could provide key features about the F-wave response without requiring manual detection of the response. Here, we use a large dataset of F-wave responses to train a model based on time–frequency characteristics to predict ALS diagnosis for patients and their anticipated survival. We applied the wavelet transform to extract temporal and spectral features from the F-wave response, which has been successful in other medical applications, such as classifying different abnormalities from ECG signals.^20,21^ The findings presented here demonstrate the efficacy of a new application of AI modelling for aiding the diagnosis and prognosis of such a complex neurological disease.

## Materials and methods

### Subjects

All procedures and protocols of this study were approved by the Mayo Clinic Institutional Review Board. We conducted a retrospective study of 46 802 patients who came to the Mayo Clinic for clinically indicated F-wave sessions between March 2003 and May 2022. Patients were included in this study if they had a diagnosis of ALS, motor neuron disease (MND) or no abnormality based on the results of their F-wave session. Throughout this manuscript, any patient who had no abnormality detected after completing the full electrodiagnostic study (including clinically indicated nerve conduction studies and needle EMG) were labelled controls. The following nerve–muscle pairs were stimulated/recorded: (i) fibular–extensor digitorum brevis; (ii) median–abductor pollicis brevis; (iii) tibial–abductor hallucis; and (iv) ulnar–abductor digiti minimi. The cohort was split into a training set (45 424 patients) and a test set (1378 patients). In the training set, 5329 patients had an EMG diagnosis code of MND, of which ALS is the most common clinical diagnosis. The remaining patients had no abnormal findings from their electrodiagnostic study results and were categorized as controls. In the test set, 689 patients confirmed to have ALS meeting Gold Coast Criteria were combined with 689 age- and sex-matched controls. Age, sex and body mass index (BMI) at the time of the F-wave session were included as features for training the model. The test set was designed to ensure that all participants were confirmed to have ALS meeting Gold Coast criteria through detailed clinical phenotyping. Owing to the size and nature of the full dataset, it was not feasible to phenotype every MND patient; however, this test set still provided an accurate representation of a representative population who would come to the Mayo Clinic for evaluation. As an exploratory analysis, we also compared the model performance on 282 patients diagnosed with inclusion body myositis (IBM) based on diagnostic information in the electrodiagnostic database or confirmed via pathology, 193 patients with cervical radiculopathy based on EMG diagnosis, 755 patients with lumbar radiculopathy based on EMG diagnosis, and 14 574 patients with peripheral neuropathy based on EMG diagnosis.

For ALS patients in the test set, additional information was collected, including the site of symptom onset, family history of neurodegenerative disease (ALS, frontotemporal dementia or parkinsonism), date of onset and date of death (if occurred). Dates of death were collected via the electronic medical record and a commercial entity (Accurint). These data were used in a Cox proportional hazards (CPH) survival analysis to identify factors decreasing survival.

### F-wave waveform preprocessing and wavelet analyses

F-wave waveforms were recorded using either Nicolet Viking Select (Natus) or Cadwell Sierra Summit (Cadwell Industries) EMG machines. Stimulation amplitude, stimulation duration, sampling rate (10–40 kHz) and recording duration were recorded for each F-wave stimulation. Each F-wave time series was resampled to 10 kHz and trimmed from 2.5 ms (to avoid the trailing edge of the stimulation artefact) to 300 ms (after which no F-wave responses were expected).

Analyses and preprocessing were performed using Python. Discrete wavelet transforms were calculated using the pywavelets toolbox.^22^ For each F-wave waveform (Fig. 1), wavelet coefficients were calculated (five-level decomposition, Daubechies 2 family). Metrics calculated for each wavelet coefficient (where $x_{c}$ is the $c^{\mathrm{th}}$ coefficient) were as follows: (i) mean [$\mu_{c}=\;\sum x_{c}/\mathrm{length}(x_{c})$]; (ii) variance [$\sigma_{c}^{2}=\sum (x_{c}-\mu_{c}{)}^{2}/\mathrm{length}(x_{c})$]; (iii) root-mean-squared [$rms_{c}=\sum \sqrt{x_{c}^{2}}/\mathrm{length}(x_{c})$]; (iv) 5th percentile; (v) 95th percentile; (vi) skewness; (vii) kurtosis; (viii) number of times the coefficient crossed the mean; (ix) relative power ($r_{c}=\frac{\;p_{c}}{\sum_{c}\;p_{c}}$); and (x) spectral entropy ($SE_{c}=-r_{c}\log\;r_{c}$). Including the stimulation duration and amplitude resulted in 52 stimulation-level features per F-wave waveform (10 features × 5 coefficients + duration + amplitude). Five to eight stimulations of a target nerve were recorded per session. Amplitude and duration were fixed across stimulations after establishing levels evoking supramaximal responses in a standard nerve conduction study preceding the F-wave study. All physicians and technicians were trained to follow established protocols and procedures for conducting the F-wave sessions to ensure that supramaximal responses were attained prior to starting the F-wave study. Although some inter-rater variability might occur, across such a large dataset these effects are expected to be minor. Means and standard deviations (SDs) of stimulation-level features were calculated for each session, resulting in 416 session-level features (4 nerves × 52 stimulation-level features × 2). Adding sex, age, BMI and the recording source (Cadwell/Natus) resulted in 420 session-level features. For nerves that were not stimulated, values were imputed using a *K*-nearest neighbour imputer with 10 neighbours.^23^ Numerical features were standard scaled. Categorical features were one-hot encoded.

To assess the contribution of the M-wave potential and F-wave potential in model performance, two additional datasets were created. The full F-wave response was divided based on the nerve stimulated to separate the segment consisting of the M-wave from the F-wave. The cut points were selected based on table 19.1 written by Laughlin,^8^ in which ulnar and median nerves were separated at 20 ms and tibial and fibular nerves at 35 ms. Wavelet features were extracted during the M-wave and F-wave segments as described above.

Some F-wave sessions had clinical annotations corresponding to key latencies and amplitudes (Fig. 1), specifically: (i) M-wave onset; (ii) M-wave negative peak; (iii) M-wave positive peak; (iv) M-wave offset; (v) F-wave onset; (vi) F-wave negative peak; (vii) F-wave positive peak; and (viii) F-wave offset. Another model was trained using these annotations to compare against the wavelet-based model.

### Modelling approach

The H2O AutoML Python package^24^ was used to train 20 models to find the model maximizing the area under the precision-recall curve (PR-AUC). This metric was selected to maximize correctly classifying ALS patients (true positives) while minimizing false negatives. The AutoML package allows for testing numerous types of machine learning models without needing to select a specific model *a priori* based on assumptions about the structure of the dataset. The following model types were trained: distributed random forest and extremely randomized trees models; generalized linear model with regularization; XGBoost gradient boosting machine (GBM); H2O GBM; and deep learning fully connected multi-layer artificial neural network. Stacking ensembles (combinations of individual models) were excluded to simplify interpretation of model performance. The PR-AUC metric was calculated based on 5-fold cross-validation on the training set to use five different train and validation sets for evaluating model performance. The model with the greatest PR-AUC score was selected for subsequent analysis.

The ultimate goal of the model presented here is to provide probabilities to a clinician to assist with making a diagnosis (rather than making a diagnosis itself). However, to assess the trained model performance, we measured the recall, precision and accuracy of the model when making predictions on the test set. Recall measured how often the model correctly classified ALS patients from the total number of ALS patients (true positives/true positives + false negatives). Precision measured how often the model correctly classified ALS patients from the total number of patients classified as ALS (true positive/true positive + false positive). Accuracy measured how often the model correctly classified ALS and control patients (true positives + true negatives/total patients). The area under the receiver operator characteristic curve (ROC-AUC) and PR-AUC provided additional measures of how well the model trained. For comparing ALS versus controls, the decision threshold was set to the incidence of MND patients in the training set (0.12) to increase recall performance. For all other comparisons, the threshold was kept at 0.5. Each metric was bootstrapped 10 000 times to obtain 5th and 95th percentiles.

Feature importance was estimated using built-in H2O functions to calculate Shapley values,^25^ and measured separately for each nerve owing to similar features being important across nerves. Pearson correlation coefficients were calculated between wavelet and annotation features to determine the similarity between feature sets.

### Survival analysis

Survival analysis was performed on the test set using CPH regression to determine factors significantly increasing risk of death. Sex, site of onset, diagnostic delay, family history, age at onset, BMI and model score for ALS patients in the test set were included in the CPH regression. Model score was defined as the log-odds of the probabilities the model classified the patient as ALS:


(1) $$\mathrm{score}=\log\;\left(\frac{\mathrm{pro}b_{\mathrm{ALS}}}{\mathrm{pro}b_{\mathrm{control}}}\right)$$

The lifelines Python package was used to perform the CPH regression.^26^ For patients with confirmed dates of death (event set to True), durations were calculated from the date of symptom onset to the date of death. For patients without a confirmed date of death (event set to False), these were considered right-censored data, and the duration was calculated from the date of symptom onset to 31 January 2023 (the last date when data were updated). Categorical features (sex, site of onset and family history) were one-hot encoded, and numerical features (age at onset, BMI and model score) were standard scaled. CPH hazard-ratio coefficients with significant *P*-values (*P* < 0.05) were identified as factors increasing risk of death.

### Statistical analyses

A *t*-test was used to compare bootstrapped model performance for the wavelet-based and annotations-based models. For the CPH regression, the lifelines toolbox performs a Wald test of a single parameter (χ^2^ distribution with d.f. = 1).^26,27^ CPH regression coefficients with *P*-values < 0.05 were used to identify significant factors that decreased survival. ANOVA with Tukey’s HSD *post hoc* tests were performed on the distributions of the model scores when comparing model predictions from the ALS patients, IBM patients, cervical and lumbar radiculopathies, peripheral neuropathies and controls with no abnormality.

## Results

### Model trained on wavelet-based features from F-wave data accurately classified ALS and control patients

Age, sex and BMI are factors that have been shown to affect survival of ALS patients^28-30^ and were included in training the model (Table 1). The training set consisted of 5329 MND patients (42.8% female) and 40 095 controls (66.5% female). For the MND patients in the training set, median age was 63.7 years [38.9, 80.1 for the 5th and 9th percentiles, respectively] and BMI was 26.0 kg/m^2^ [19.2, 37.2]; for the control patients, median age was 50.7 [24.8, 76.6] years and BMI was 26.8 [19.5, 40.0] kg/m^2^. The test set was age and sex matched, with 689 patients in each (43.7% female). Although BMI was not specifically controlled for in the test set, the median BMI was 26.2 [19.2, 37.7] kg/m^2^ for ALS and 27.5 [20.8, 39.1] kg/m^2^ for controls.

**Table 1:** Description of the demographics of the training set and test set

Characteristic	Training set		Test set	
	MND	Controls	ALS	Controls
*n*	5329	40 095	689	689
Sex (female)	2280	26 657	301	301
Sex (male)	3049	13 438	388	388
Age^a^ (years)	63.7 [38.9, 80.1]	50.7 [24.8, 76.6]	64.7 [42.5, 80.3]	64.7 [42.5, 80.3]
BMI^a^ (kg/m^2^)	26.0 [19.2, 37.2]	26.8 [19.5, 40.0]	26.2 [19.2, 37.7]	27.5 [20.8, 39.1]

ALS = amyotrophic lateral sclerosis; BMI = body mass index; MND = motor neuron disease.

^a^Age and BMI are reported as 50th [5th, 95th] percentiles.

For both the training set and the test set, patients had different nerves stimulated (depicted with UpSet plots in Fig. 1B and C). The relative numbers of sessions in which the ulnar, fibular and tibial nerves were stimulated were similar for each set. For both sets, the median nerve was the least stimulated and the pair of the fibular and tibial nerves was the most often stimulated. Features were imputed for nerves that were not stimulated as described in the Materials and methods.

**Figure 2:**
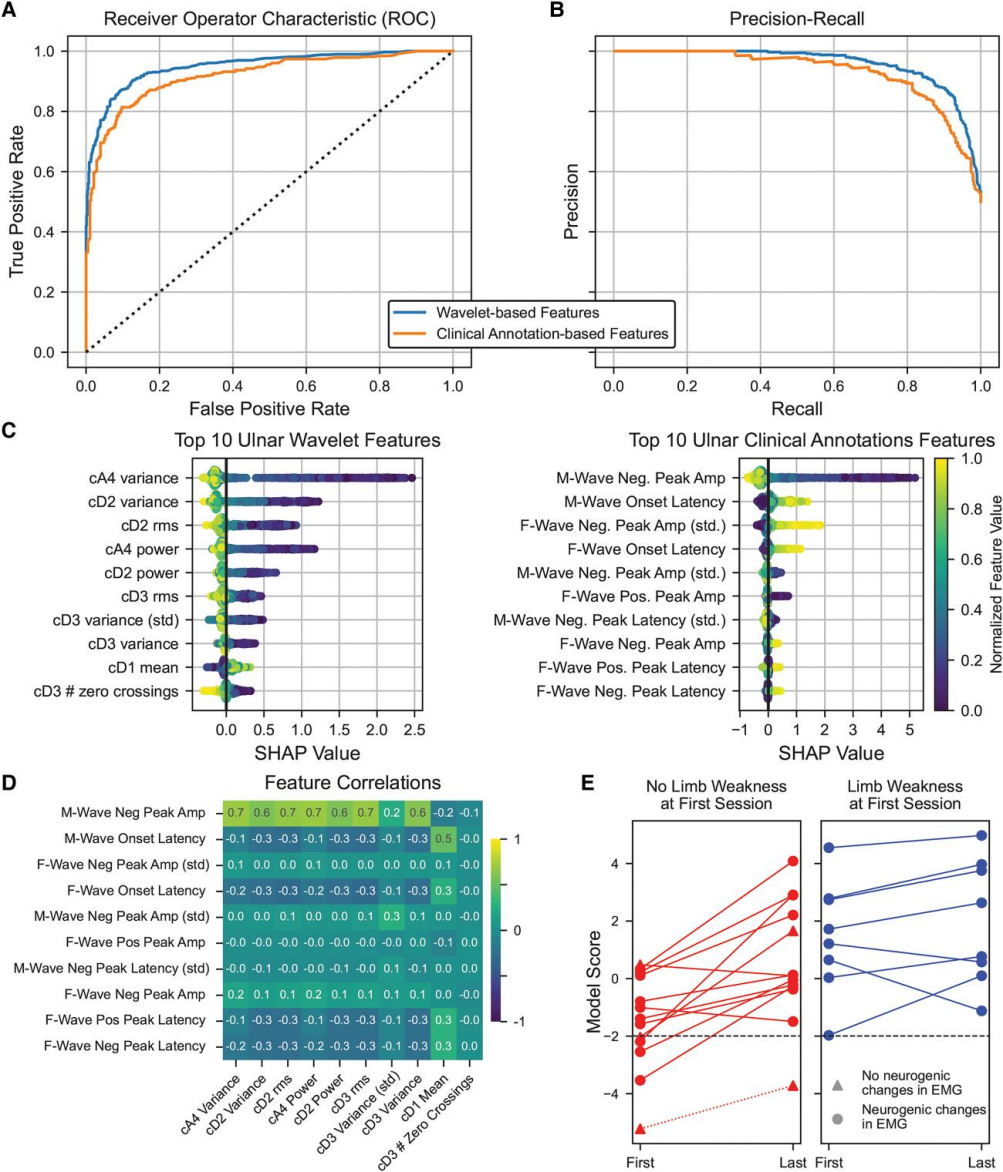
**Comparing wavelet- and clinical annotation-based models.** (**A**) Receiver operator curves. (**B**) Precision-recall curves. Dashed black lines indicate chance levels. (**C**) Feature importance for top 10 ulnar wavelet features and clinical annotation features. Wavelet coefficients are represented in a similar manner to Fig. 1. Features with (std) represent the standard deviation across trials in the F-wave session. (**D**) Correlation matrix between the top 10 wavelet features (*x*-axis) and clinical annotations (*y*-axis). Features are sorted by Shapley Additive Explanations (SHAP) value as shown in **C**. Pearson correlations are colour coded from −1 to 1, with yellow boxes indicating positive correlations and blue indicating negative correlations. (**E**) Comparison of model scores between bulbar onset patients who had no limb weakness at the first F-wave session but limb weakness at the last session versus bulbar onset patients with weakness present at both sessions. Filled circles indicate that neurogenic changes were detected in EMG activity during the session. Filled triangles indicate that no neurogenic changes were detected in EMG activity during the session. One patient had no neurogenic changes in EMG activity during both sessions (dashed line).

After running the H2O AutoML function on wavelet features extracted from the full waveform, the GBM model was selected based on its cross-validated PR-AUC score of 0.86 on the training set (scores ranged from 0.78 to 0.86 across models tested). The ROC curve and precision-recall curve (Fig. 2A and B) provide a visualization of how well the model performed at different decision thresholds on the test set, with an ROC-AUC of 0.95 and a PR-AUC of 0.96. Table 2 shows the bootstrapped performance of the model when using the MND prevalence rate of 0.12 from the training set as the decision threshold. The model had a recall of 0.91 at correctly classifying ALS patients and minimizing false negatives. Additionally, the model had a mean precision of 0.87, suggesting that the model also minimized false positives to maximize recall performance. Model accuracy of classifying the ALS and control patients was 0.89, demonstrating that the model performed well at differentiating between ALS and controls.

**Table 2:** Bootstrapped model performance on the test set

Parameter	Wavelet-based model(mean ± SD)	Clinical annotations-based model(mean ± SD)
Recall	0.91 ± 0.01	0.87 ± 0.02
Precision	0.87 ± 0.01	0.83 ± 0.02
Accuracy	0.89 ± 0.01	0.85 ± 0.01
ROC-AUC	0.95 ± 0.01	0.92 ± 0.01
PR-AUC	0.96 ± 0.01	0.93 ± 0.01

Each metric was significantly different between the wavelet-based model and the clinical annotations-based model (*t*-test, *P* < 0.01). ROC-AUC = area under the receiver operator characteristic curve; PR-AUC = area under the precision-recall curve; SD = standard deviation.

### Wavelet-based features outperformed clinical annotation-based features

We trained a comparable model by using clinical annotations of the F-wave waveform as features. The clinical annotations identified key amplitudes and latencies of the F-wave waveform, such as negative and positive peaks. We used the same GBM model architecture and trained it on 11 649 F-wave sessions with F-wave annotations. The resulting model was tested against 762 F-wave sessions (381 ALS patients and 381 age/sex-matched controls). The cursor-based model also performed well on the test set, based on the bootstrapped model performance shown in Table 2. Both feature sets performed well, but the wavelet-based model performed better in terms of recall (0.91 versus 0.87), precision (0.87 versus 0.83) and accuracy (0.89 versus 0.85) compared with the annotations-based model. When examining the ROC and precision-recall curves (Fig. 2A and B), the ROC-AUC was 0.92 and PR-AUC was 0.93 for the clinical annotations, demonstrating that even if the decision threshold was changed, the wavelet-based features would perform slightly better than the clinical annotations.

We next examined the feature importance of both models using Shapley Additive Explanations (SHAP).^25^ The pattern of important features was similar for each nerve; therefore, we have depicted the importance of ulnar nerve features (Fig. 2C; the remaining nerves are in Supplementary Fig. 1). Wavelet features measuring variance, root-mean-square and skewness of the wavelet coefficient were identified as most important. For the clinical annotations, negative peak amplitude, onset latency and ending latency of the M-wave and F-wave were most important. We calculated the correlation matrix between these wavelet and clinical annotation features to determine the similarity of the information encoded (Fig. 2D). Both features were sorted based on their SHAP values, and there are some strong positive and negative correlations (e.g. M-wave negative peak amplitude with variance and root-mean-square of numerous wavelets). However, there was no obvious one-to-one mapping, suggesting that the wavelet features might be encoding additional or different information from the clinical annotations.

### Assessing the contributions of the M- and F-waves

**Figure 3:**
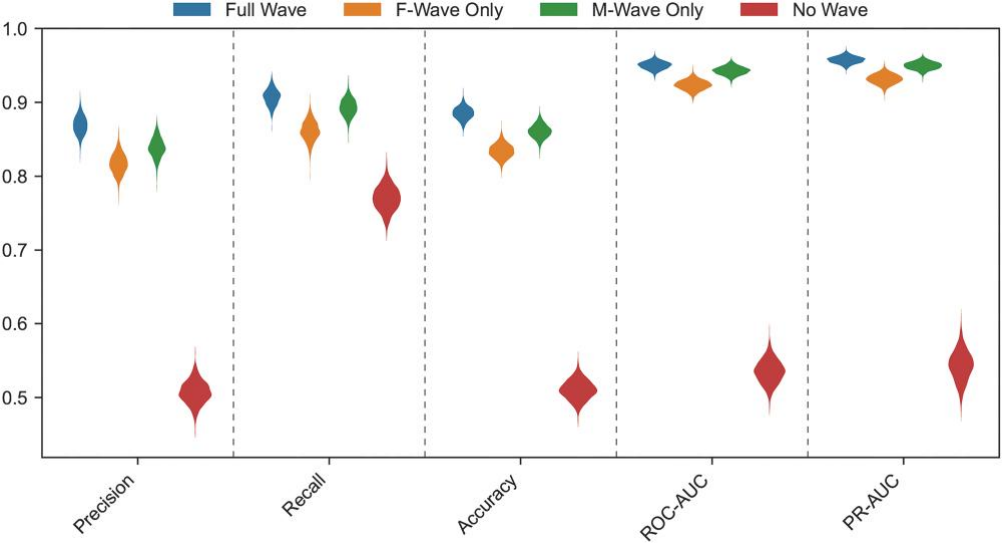
**Comparison of the model performance metrics when using different segments of the post-stimulus waveform to train the model.** The distribution of each bootstrapped metric is displayed using a violin plot. The full wave (consisting of both the M- and F-wave potentials) is depicted in blue, the F-wave-only potential is depicted in orange, the M-wave only-potential in green, and performance when no wavelet information is used (only training on age, sex, body mass index and session source) is depicted in red. ROC-AUC = area under the receiver operator characteristic curve; PR-AUC = area under the precision-recall curve. Precision, recall and accuracy were assessed at the same 0.12 decision threshold as used for the metrics in Table 2.

The model results shown in Table 2 extracted wavelet-based features from the entire post-stimulus waveform response, which consists of both the M-wave and F-wave potentials (as depicted in Fig. 1A). How much does each potential contribute to the overall performance of the model? To assess this, we trained two additional models, in which the wavelet features were extracted from only the M-wave potential or only the F-wave potential. The model performance from these models was then compared against the model presented in Table 2 (labelled the full wave model). As depicted in Fig. 3, precision, recall, accuracy, ROC-AUC and PR-AUC of the full wave (depicted in blue) outperformed the F-wave-only model (orange) and the M-wave-only model (green). As a control, we trained a fourth model using only demographic information (age, sex, BMI and session source; depicted in red). Both the M-wave-only and F-wave-only models performed much better than this ‘no wave’ model for each of the performance metrics, confirming that the wavelet-based models were extracting important features from the post-stimulus response to differentiate ALS patients from patients with no abnormality. The M-wave-only model outperformed the F-wave-only model for each of the metrics in Fig. 3. Based on the feature importance plots in Fig. 2C or Supplementary Fig. 1, spectral information from both the M-wave and the F-wave (e.g. variance, power) were important features for the full wave model, probably contributing to its improved performance over the M-wave-only and F-wave-only models.

### Model score is a risk factor for death in ALS patients

**Figure 4:**
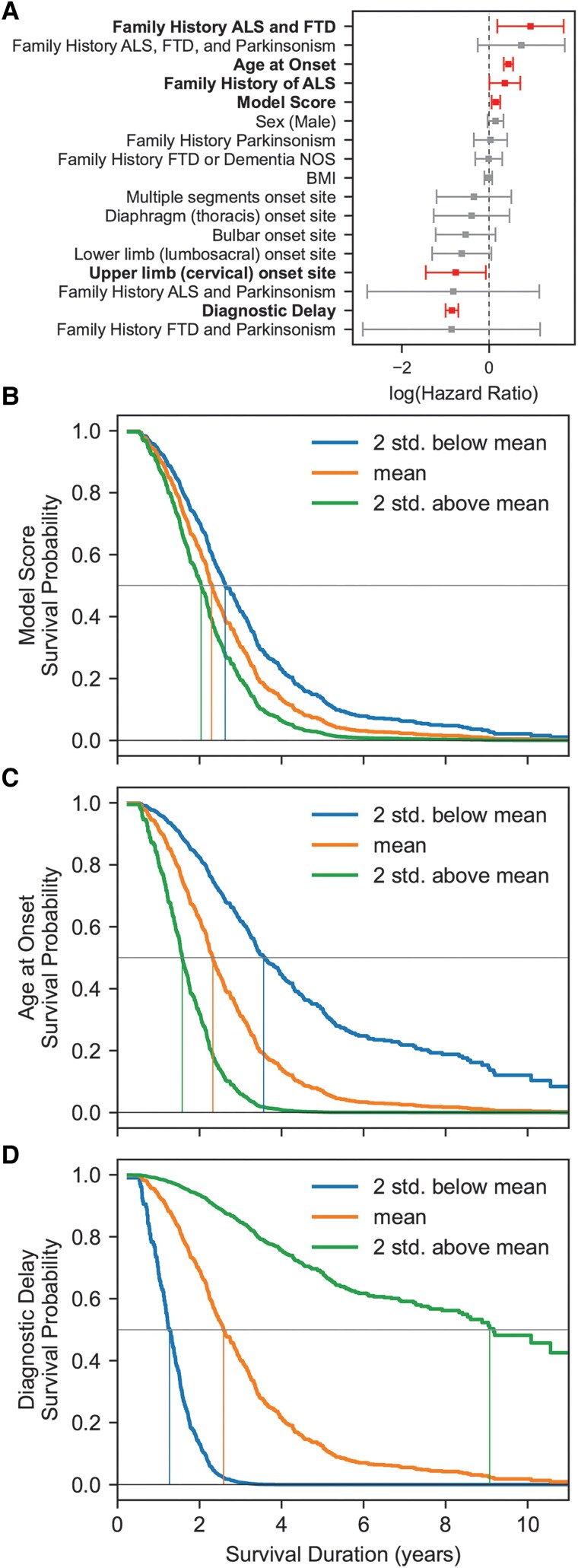
**Model score and age at onset are factors affecting survival duration.** (**A**) The 95% confidence intervals for each Cox proportional hazards coefficient. Significant factors are indicated with bold, and box plots are highlighted in red. (**B**) Effect of model score on survival probability. (**C**) Effect of age at onset on survival probability. (**D**) Effect of diagnostic delay on survival probability. Blue, orange and green traces show survival probability at 2 SD below the mean, the mean and 2 SD above the mean value of the model score, age at onset or diagnostic delay. Vertical thin lines indicate the median survival. The thin grey horizontal line indicates 50% survival probability. ALS = amyotrophic lateral sclerosis; BMI = body mass index; FTD = frontotemporal dementia; MND = motor neuron disease; NOS = not otherwise specified; SD = standard deviation.

CPH survival analysis was performed on the ALS patients in the test set to determine which factors affect survival. The following factors were included in the survival analysis: family history, site of onset, diagnostic delay, age at onset, sex, BMI and the wavelet-based model score. Age at onset, the model score and family history of ALS alone or with frontotemporal dementia were the only statistically significant factors that decreased survival (Fig. 4A). Diagnostic delay and upper limb onset site were the only statistically significant factors that increased survival.

We examined the effect of the score further by estimating the partial effect of survival when this variable changed (but keeping all variables constant) in the CPH model (Fig. 4B). Individuals with a score 2 SDs above the mean (27 months) had a survival of 24 months, whereas individuals with a score 2 SDs below the mean had a survival of 31 months. For age at onset (Fig. 4C), individuals 2 SDs above the mean had a survival of 19 months, whereas those 2 SDs below the mean had a survival of 43 months. Diagnostic delay increased survival risk (Fig. 4D), in which individuals 2 SDs above the mean had a survival of 109 months, whereas those 2 SDs below the mean had a survival of 15 months. Family history of ALS or ALS and frontotemporal dementia and upper limb onset were also identified as significant risk indicators. Patients who had a history of ALS had a survival of 24 months, individuals with a history of ALS and frontotemporal dementia had a survival of 18 months, and those with upper limb onset site had a survival of 40 months (in comparison to 27 months for those with no family history of ALS or ALS and frontotemporal dementia or no upper limb onset site). Thus, the time at which a patient starts to develop symptoms, the delay in diagnosing these symptoms, the model score based on F-wave data, a family history of certain neurodegenerative diseases and upper limb onset each plays an independent role in the estimated survival of a patient.

### Exploratory analysis of bulbar onset patients

Bulbar onset in ALS has been associated with a more rapid disease progression in comparison to other ALS subtypes and can be more challenging to diagnose early in the disease.^29,31,32^ Being able to identify risks for bulbar onset ALS sooner could greatly improve clinical management for such patients. As an exploratory analysis, we examined the model scores for a subset of 21 ALS bulbar onset patients whose disease started with predominantly speech or swallowing impairment and had multiple F-wave sessions (Fig. 2E). In this cohort, there might not be initial clinically evident limb weakness, hence the role of limb-generated F-waves is unclear. Of the 21 patients, 13 had no limb weakness at this first F-wave session (left panel, red traces). Three of these 13 patients also did not have any neurogenic changes recorded in their first needle EMG session (triangles in Fig. 2E). The model predicted that 8 of the 13 patients probably had ALS based on the initial F-wave responses. By the final F-wave session, 12 of 13 patients were predicted to have ALS based on the model score. The patient who was not predicted to have ALS (indicated by the dashed line in Fig. 2E) had no limb weakness or neurogenic changes during any F-wave session but developed limb weakness at a later visit, when F-Waves were not performed. For the eight patients who presented with limb weakness at their first F-wave session, the model predicted that all of them probably had ALS based on the initial F-wave responses (Fig. 2E, right panel, blue traces) and also their final F-wave responses. Because bulbar onset patients can be more difficult to diagnose and have shorter survival durations,^32^ detecting ALS sooner could improve clinical management and potentially enable inclusion in experimental therapies to try to mitigate the adverse effects of the disease.

### Model performance on diagnoses mimicking ALS

**Figure 5:**
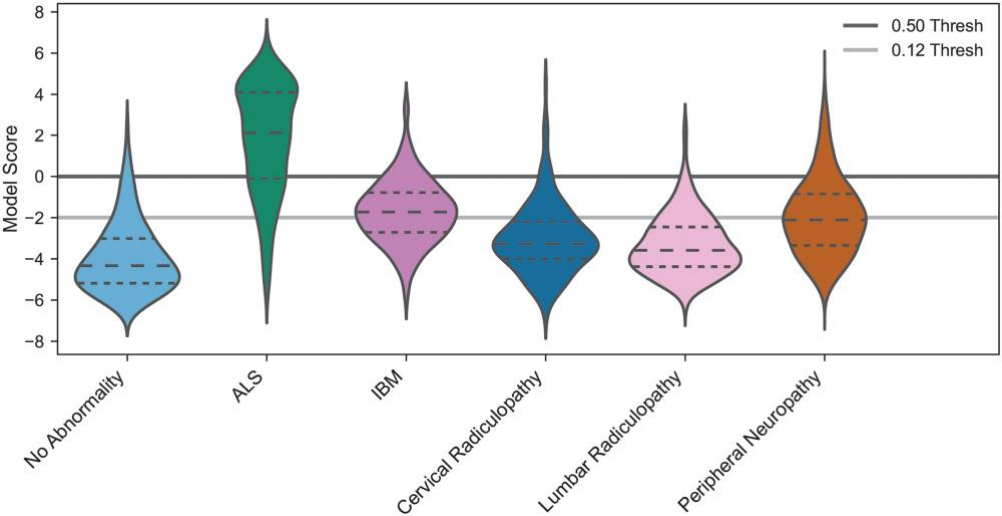
**Model score distributions differentiate patients with ALS from no abnormality and other mimics.** Distributions of model scores are shown for patients with no abnormality (light blue), ALS patients (green), IBM patients (purple), cervical radiculopathy patients (dark blue), lumbar radiculopathy patients (pink) and peripheral neuropathy patients (brown). Violin plots depict the median (dashed lines) and 25th and 75th percentiles (dotted lines). The light grey horizontal line corresponds to the 0.12 decision threshold when using the prevalence rate of motor neuron disease in the training set. The darker grey horizontal line corresponds to using a 0.50 decision threshold typically used for binary classifiers. Where the decision threshold is placed affects the precision and recall of the model; however, the ALS distribution is statistically different from all other groups (*P* < 1 × 10^−5^, ANOVA, Tukey’s HSD *post hoc* test). ALS = amyotrophic lateral sclerosis; IBM = inclusion body myositis.

As an additional exploratory analysis, we examined the model scores when processing F-wave responses from patients with diagnoses that might be confused with ALS. These included patients with IBM, cervical radiculopathy, lumbar radiculopathy, peripheral neuropathy or controls (in the original test set). As depicted in Fig. 5, the model score distribution for the ALS cohort is significantly different from each of the other diagnoses and these diagnoses were also statistically different from one another (*P* < 1 × 10^−5^, ANOVA, Tukey’s HSD *post hoc* test). At the 0.12 threshold used previously, the model correctly identified 90.6% (624/689) of ALS patients as having ALS, 86.5% (596/689) of patients with no abnormality, 43.2% (121/280) of IBM patients, 78.2% (151/193) of cervical radiculopathy patients, 82.3% (621/755) of lumbar radiculopathy patients and 52.5% (7648/14574) of peripheral neuropathy patients as not having ALS. If the threshold was set to 0.5 (a typical default threshold for binary classifiers), the model correctly identified 73.6% (507/689) of ALS patients as having ALS and 96.8% (667/689) of controls, 88.6% (248/280) of IBM patients, 93.8% (181/193) of cervical radiculopathy patients, 97.5% (736/755) of lumbar radiculopathy patients and 85.8% (12500/14574) of peripheral neuropathy patients as not having ALS. In this scenario, more ALS patients were missed, but fewer patients with mimicking presentations were misidentified as having ALS. Overall, the model does well at differentiating between the groups depending on the desired decision threshold, especially considering that the model was trained to differentiate only between ALS cases and controls. Clinically, the overall goal of our model is to provide physicians with probabilities that a patient might have ALS that they can use when making their final diagnosis. Based on the results shown in Fig. 5, a model using F-wave responses could be a viable solution for achieving such a goal.

## Discussion

In the present study, we have used machine learning/AI approaches to interrogate a large clinical database of nerve conduction F-waves from >46 000 patients at a tertiary centre. We analysed F-wave responses owing to the complexity of the evoked motor unit responses and their multiple measurable waveform parameters, in contrast to a standard compound muscle action potential. Although the electrodiagnostic study used here is called an ‘F-wave study,’ the post-stimulus response consisted of both the M-wave and F-wave compound action potentials. However, as shown in Fig. 3, incorporating features from both the M-wave and the F-wave yielded better model performance than relying only on the standard M-wave compound muscle action potential. Although the variable morphology of F-wave responses might be viewed by some as a limit to their interpretability, AI models can use this variability to extract important data features that can improve model performance.^33^ Here, we applied a data-driven AI approach to nerve conduction F-waves, which yielded a model that was able to: (i) discern ALS from normal; (ii) predict ALS survival; (iii) help to predict those with bulbar symptoms that might progress to widespread ALS; and (iv) provide probabilities for the diagnosis of ALS, IBM, cervical or lumbar radiculopathy, peripheral neuropathy or controls. We contend that this approach has broad potential to benefit ALS diagnosis, prognosis and clinical trial design.

With advances in AI techniques, significant efforts are being made to incorporate these models into the clinical workflow. However, developing AI models using clinical data specific to the clinical disease of interest is important to understand how the model is performing. Here, we demonstrate that AI models trained on electrodiagnostic data performed well at predicting the likelihood of ALS and survival risks. Wavelet-based features from the F-wave response provided a data-driven feature set based on data already being collected clinically. Importantly, the wavelet-based model outperformed a similar model trained on clinical annotations of the F-wave response. Thus, a wavelet-based approach could provide a data-driven alternative to manually identifying F-wave characteristics. This approach could enable clinicians to spend less time annotating data and more time working directly with the patients.

One concern when training AI models is making sure that the model performs well when encountering data outside of what was used in the training set. For the model presented here, the data used to train the model involved patients with MNDs and no abnormalities; however, patients with symptoms that mimic ALS could significantly reduce the impact of such a model. To address this, we performed an exploratory analysis, in which we used F-wave responses from patients with IBM, cervical radiculopathy, lumbar radiculopathy or peripheral neuropathy, which can mimic the symptoms of ALS. Although the model was not trained on data from these patients, the model scores shown in Fig. 5 demonstrate that there is separation between patients with ALS and all the other disease groups. This finding is promising, because it suggests that electrodiagnostic data are useful for training AI models to help with differentiating between different neurological diseases.

Previous biomarkers have been described using specific, observable parameters of nerve conduction responses for classifying patients with ALS. For example, the NI used the frequency of observing an F-wave response, the amplitude of the M-wave and the latency of the M-wave as parameters for calculating the metric.^11,14,16,17^ Here, we took a more data-driven approach that incorporates these parameters but also additional temporal and spectral information via wavelet transformations of the F-wave response. This approach demonstrated that these features outperformed a model that used clinical annotations of key latencies and amplitudes of the F-wave response. By transforming the response into time–frequency characteristics, the interpretability of how the most important features relate to the F-wave response becomes a little difficult to describe. Figure 2D and Supplementary Fig. 1 depict how the top 10 wavelet features strongly correlated with the amplitude of the M-wave but also with the other clinical annotations. Thus, the wavelet transformation might have provided additional information from the F-wave response beyond those more easily identified from key latencies and amplitudes. AI approaches often take advantage of extracting important information that is not easily observed visually.^33^ This has been demonstrated in other fields, such as cardiology, where AI models detected atrial fibrillation based on subtle changes in a brief recording of the sinus rhythm that would have been harder to identify from visual inspection.^34^ Here, the wavelet features might detect a similar subtle change in the F-wave response, making it challenging to even the expert eye to detect visually what specific waveform characteristics are driving the prediction of the model. Future analyses could help to correlate underlying neural mechanisms with most important wavelet features of the model to provide guidance to clinicians about what to focus on when inspecting F-wave responses visually.

Numerous factors have been attributed to shortened survival in ALS patients, including sex, BMI, bulbar onset and a positive family history of ALS.^28-30^ Here, the F-wave model score was identified as a factor predicting survival risk, along with onset age, diagnostic delay, upper limb onset site and family history of ALS or ALS and frontotemporal dementia. In an exploratory subset of bulbar onset patients, the model accurately predicted the likelihood of ALS before limb weakness was detected clinically. Similar biomarkers have been proposed for detecting ALS based on electrodiagnostic data^11,18,19^; however, to our knowledge this is one of the first data-driven approaches on such a large database of F-wave responses. Given that this was a retrospective study, future prospective studies should incorporate these data-driven factors with prognostic or clinical factors (e.g. Revised Amyotrophic Lateral Sclerosis Functional Rating Scale scores, forced vital capacity and genetic factors) to gain a better understanding of how each factor affects the disease time course for each patient.

To date, there are limited interventions for modifying the disease course of ALS.^35^ ALS disease progression is heterogeneous, with some patients having faster disease progression than others. This heterogeneity can lead to difficulty in developing clinical trials with appropriate patient stratification.^36^ AI approaches have been proposed to improve the stratification of patients in clinical trials and to predict disease progression.^37^ However, there is still a need to diagnose ALS sooner, to allow more patients to be eligible for enrolment in clinical trials and inform patients about how long they can expect the disease course to last.^38^ Data-driven approaches could lead to crucial biomarkers for clinical trial end points to determine the efficacy of experimental interventions for treating ALS.

Many studies of ALS use traditional demographic information and diagnostic information such as Revised Amyotrophic Lateral Sclerosis Functional Rating Scale scores for prognosing disease course.^39^ Tools that use data from modalities such as electrodiagnostic data could help with the prognosis and diagnosis of ALS.^37^ Numerous biomarkers have been proposed previously to use electrodiagnostic data for predicting ALS disease progression^11,12^; however, the rate of the disease progression can make development of such a biomarker challenging.^14^ The findings presented here are not intended to be a ‘final model’ but instead a demonstration of how data-driven features extracted from the time series of the F-wave response might improve diagnoses and prognoses of ALS. Future iterations can expand on this approach to develop a model that includes additional mimics in the training set to improve overall performance. However, the ultimate goal is not to have the model itself make the clinical diagnosis, but instead to provide probabilities for a clinician to use to make the final diagnosis themselves. Overall, using an AI model to provide information about survival risk and early diagnosis is an important step towards development of a clinical tool to help manage such a rapidly progressing disease.

## Supplementary Material

awaf014_Supplementary_Data

## Data Availability

Data are available on request owing to privacy/ethical restrictions.
