# Supplementary material for: Artificial intelligence models using F-wave responses predict amyotrophic lateral sclerosis
Source: Brain. 2025 Jan 16;148(7):2320–30. doi: 10.1093/brain/awaf014 (PMC12233553; doi:10.1093/brain/awaf014)
Supplement: awaf014_Supplementary_Data [file awaf014_supplementary_data.pdf]

Supplementary material

Supplementary Figure 1 Feature importance of each nerve pair and feature correlations.

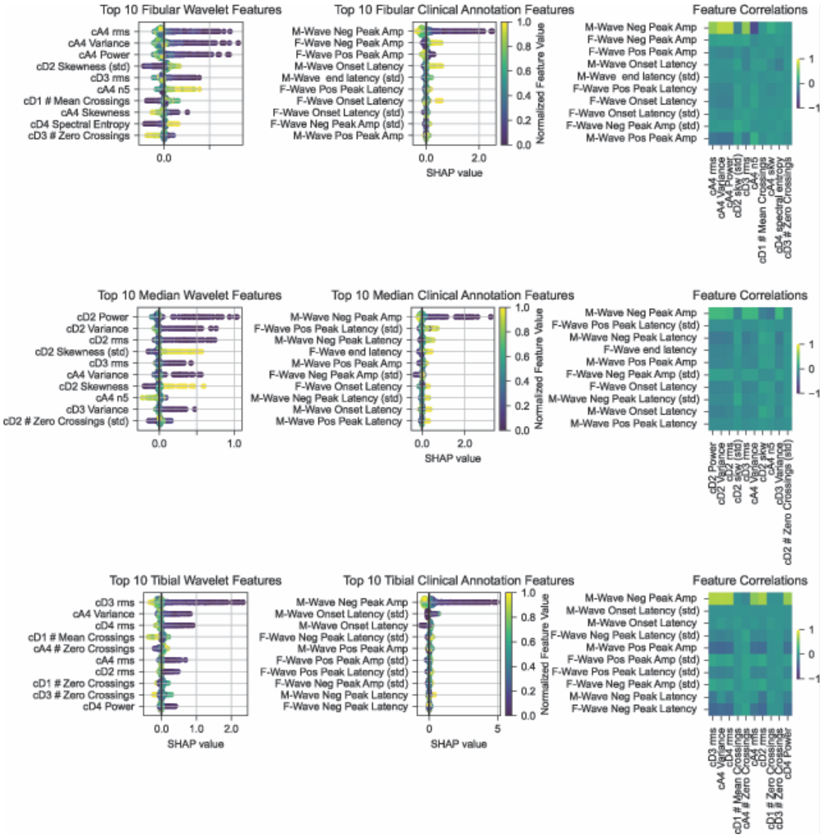

Feature importance and correlations between wavelet-based features and clinical annotations-based features are similar for the Fibular, Median, and Tibial nerves. Conventions consistent with Fig. 2.
